# Better cardiovascular outcomes of type 2 diabetic patients treated with GLP-1 receptor agonists versus DPP-4 inhibitors in clinical practice

**DOI:** 10.1186/s12933-020-01049-w

**Published:** 2020-06-10

**Authors:** Enrico Longato, Barbara Di Camillo, Giovanni Sparacino, Lara Tramontan, Angelo Avogaro, Gian Paolo Fadini

**Affiliations:** 1 Department of Information Engineering, University of Padova, 35100 Padua, Italy; 2 Arsenàl.IT, Veneto’s Research Centre for eHealth Innovation, 31100 Treviso, Italy; 3 Department of Medicine, University of Padova, Via Giustiniani 2, 35100 Padua, Italy

**Keywords:** Observational, Registry, outcome, Epidemiology, Drug therapy

## Abstract

**Background:**

Cardiovascular outcome trials in high-risk patients showed that some GLP-1 receptor agonists (GLP-1RA), but not dipeptidyl-peptidase-4 inhibitors (DPP-4i), can prevent cardiovascular events in type 2 diabetes (T2D). Since no trial has directly compared these two classes of drugs, we performed a comparative outcome analysis using real-world data.

**Methods:**

From a database of ~ 5 million people from North-East Italy, we retrospectively identified initiators of GLP-1RA or DPP-4i from 2011 to 2018. We obtained two balanced cohorts by 1:1 propensity score matching. The primary outcome was the 3-point major adverse cardiovascular events (3P-MACE; a composite of death, myocardial infarction, or stroke). 3P-MACE components and hospitalization for heart failure were secondary outcomes.

**Results:**

From 330,193 individuals with T2D, we extracted two matched cohorts of 2807 GLP-1RA and 2807 DPP-4i initiators, followed for a median of 18 months. On average, patients were 63 years old, 60% male; 15% had pre-existing cardiovascular disease. The rate of 3P-MACE was lower in patients treated with GLP-1RA compared to DPP4i (23.5 vs. 34.9 events per 1000 person-years; HR: 0.67; 95% C.I. 0.53–0.86; p = 0.002). Rates of myocardial infarction (HR 0.67; 95% C.I. 0.50–0.91; p = 0.011) and all-cause death (HR 0.58; 95% C.I. 0.35–0.96; p = 0.034) were lower among GLP-1RA initiators. The as-treated and intention-to-treat approaches yielded similar results.

**Conclusions:**

Patients initiating a GLP-1RA in clinical practice had better cardiovascular outcomes than similar patients who initiated a DPP-4i. These data strongly confirm findings from cardiovascular outcome trials in a lower risk population.

## Background

Cardiovascular complications of type 2 diabetes (T2D) remain an unmet need. Despite aggressive control of concomitant risk factors, the rate of major adverse cardiovascular events (MACE) is higher in diabetic than in non-diabetic individuals [1]. Results of cardiovascular outcome trials (CVOTs) prompted recommendations to prioritize two classes of glucose lowering medications (GLM) for secondary prevention of MACE in people with T2D, namely glucagon-like peptide-1 receptor agonists (GLP-1RA) and sodium glucose cotransporter-2 inhibitors (SGLT-2i) [2]. In addition to improving glucose control, these medications exert beneficial effects on body weight and blood pressure [3].

Most CVOTs on GLP-1RA were performed in patients with T2D and established cardiovascular disease [4]. As compared to placebo, treatment with liraglutide, semaglutide, or albiglutide reduced the risk of MACE [5–7]. In view of these strong benefits, the European Society of Cardiology guidelines have suggested that GLP-1RA may be recommended even as first-line in patients with T2D and established cardiovascular disease [8]. The REWIND study, conducted on patients with T2D, 70% of whom were free from established cardiovascular disease, found that the GLP-1RA dulaglutide reduced MACE rates compared to placebo [9]. Therefore, it is possible that the protective effects of GLP-1RA extend to T2D patients with a relatively lower cardiovascular risk.

Despite the aforementioned cardiovascular benefits and the glycemic effectiveness shown also in the real world [10, 11], GLP-1RA are still underutilized in clinical practice, in favor of other GLM that are mostly devoid of cardioprotective effects, such as dipeptidyl-peptidase-4 inhibitors (DPP-4i) [12]. The daily injectable administration regimen of some GLP-1RA has been a detrimental factor against their widespread clinical use, but even weekly GLP-1RA are being prescribed to a minority of T2D patients.

There is growing agreement that findings from clinical trials need to be verified in clinical practice using routinely accumulated clinical data [13, 14]. The experimental and “controlled” trial setting is extremely different from routine care in terms of patient selection, motivation, compliance, as well as follow-up procedures and resource availability. Also, the generalizability of CVOTs to the T2D population seen in clinical practice is questionable [15].

The association between therapy with GLP-1RA and lower rates of MACE have been confirmed in a few observational studies [16, 17], but data on the comparison with DPP-4i are scant. Also, no trial has directly compared cardiovascular outcomes of T2D patients randomized to receive a GLP-1RA or a DPP-4i, nor any is planned. In the absence of dedicated trials, observational studies can help fill such a gap.

We herein performed a retrospective study on an administrative claim database to compare cardiovascular outcomes of T2D patients who initiated a GLP-1RA or a DPP-4i on top of a prior GLM regimen.

## Methods

### Data source and cohort identification

The main data source for the present study was the administrative data repository of the Veneto Region, North East Italy. All healthcare contacts involving the Region’s ~ 5 million inhabitants are recorded to report expenditures to the central government. To complement this infrastructure, a regional Health Information Exchange (rHIE) system has been implemented for the real-time sharing of healthcare documents [18], including laboratory reports. This was a retrospective, observational study involving the entire Veneto region. The initial subject pool comprised all Italian citizens resident in the Region who, according to Veneto’s register of healthcare beneficiaries [19] had been eligible beneficiaries for at least 1 year between January 1st, 2011 and September 30th, 2018, or time of death. For each subject, we collected all available information, including exemptions from co-payment, and all administrative claims concerning prescriptions, refills, and hospitalizations (procedures and post-discharge diagnosis codes). In the absence of a centralized diabetes registry, we applied a validated claims-based algorithm with 97.6% precision, 95.7% recall, 87.9% specificity [20] in identifying citizens affected by diabetes. Among these, we selected all new initiators of GLP-1RA (exenatide, liraglutide, lixisenatide, dulaglutide) or DPP-4i (sitagliptin, vildagliptin, alogliptin, linagliptin, saxagliptin) who had started their therapies within the observation window but had not been treated with fast-acting insulin or the other drug. This exclusion criterion was applied because, in Italy, the combination of fast-acting insulin and GLP-1RA or DPP-4i was not reimbursed; in addition, even spot use of fast-acting insulin is considered a proxy of disease severity or intercurrent illness. The distinction between ongoing and newly initiated therapies was based on the presence (or absence, respectively) of prescriptions of each drug within 7 months of the first prescription of an A10-class drug in the patient’s claims. We defined the date of first appearance of either a GLP-1RA (ATC A10BJ) or a DPP-4i (A10BH, A10BD07-13, A10BD19, A10BD21, or A10BD24-25) after this 7-month period as the patient’s index date. The 7-month delay was chosen based on a sensitivity analysis comparing prescription with refill rates, showing that the vast majority of prescriptions are refilled within 7 months. In our primary, “as treated” (AT) analysis, we followed each subject from the index date until therapy discontinuation or the last available observation. In a sensitivity analysis, we followed an “intention to treat” (ITT) approach, disregarding therapy discontinuation as a censoring criterion.

Since prescription of cardioprotective drugs can reflect perception of an imminent cardiovascular events or a planned cardiovascular intervention, in order to avoid this reverse causality, we ignored all events occurring within 2 months from the index date. This delay also allows hospitalization administrative claims to appear in the repository.

### Data anonymization

All the data used in this study were previously anonymized as per the Italian law concerning their usage for research and governance purposes [18]. Based on national regulations for such studies on anonymized administrative claims, patients’ informed consent was not mandatory.

### Outcome definition

The primary outcome was a modified definition of the 3-point major adverse cardiovascular event (3P-MACE), i.e., a combination of myocardial infarction, stroke, or all-cause death. Due to the unavailability of causes of death, all-cause death was used in place of the traditional cardiovascular death within the 3P-MACE. This modification was considered acceptable because about 70% of deaths in people with diabetes are caused by cardiovascular disease [21]. Secondary endpoints were: individual components of the 3P-MACE and hospitalization for heart failure (HHF). Operatively, the presence of the following ICD-9-CM diagnosis codes in a patient’s claims denoted the occurrence of the corresponding endpoint: 410-414 myocardial infarction, 431-436 stroke, 428 hospitalization for heart failure. Due to the time resolution of anonymized dates of death, all event times were expressed in months.

### Propensity score matching and statistical analysis

We balanced GLP-1RA and DPP-4i initiators via propensity score matching (PSM), using the nearest neighbor method and the logit distance, with maximum caliper set to 0.06% of the propensity score (PS) standard deviation. The estimated PS were the output of a logistic regression model trained on patients’ characteristics, i.e., age at index date, sex, claims-based history length (months between the first available claim and the index date), claims-based diabetes duration (months between the first diabetes-related claim and the index date); pre-existing conditions, i.e., hypertension, dyslipidemia, peripheral circulatory complications, myocardial infarction, ischemic heart disease, stroke or TIA, heart failure, cardiovascular disease, neurological complications, ocular complications, renal complications, chronic kidney disease, severe hypoglycemia, chronic pulmonary disease, systemic inflammatory disease, cancer, Charlson comorbidity index [22, 23]; glucose lowering medications in the entire patient’s history, i.e., number of different A10B-class drugs (“blood glucose lowering drugs, excluding insulins”) and insulin therapy; use of glucose lowering medications in the year before the index date, including long-acting insulin, metformin, sulfonylureas, SGLT-2i, pioglitazone; and use of other drugs in the year before the index date, including ACE inhibitors, diuretics, beta blockers, other antihypertensives, statins, fibrates or omega-3, PCSK9 inhibitors, ezetimibe, and platelet aggregation inhibitors. Additional file 1: Table S1 reports the definition of these variables via administrative claims.

We tested the balance obtained by PSM using the Chi square test for dichotomous variables, and Mann–Whitney’s U test for age at index date, claims-based history length, claims-based diabetes duration, Charlson index, and number of A10B-class drugs. We defined the two cohorts to be well-balanced if all associated p-values were greater than 0.05 or the effect size ware sufficiently small (standardized mean difference between − 0.10 and 0.10). Laboratory data were available for a limited subset of subject. Hence, following a previously published approach [24], we verified whether good balance in administrative claims would translate into good balance in the laboratory data closest to the index date. The criteria for this balance assessment were the same as in the previous evaluation (p > 0.05 or absolute SMD < 0.10). Laboratory variables were fasting glucose, HbA1c, total cholesterol, HDL cholesterol, LDL cholesterol, triglycerides, eGFR (CKD-EPI formula [25]). Systolic blood pressure and diastolic blood pressure were also recorded, when available.

In our primary analysis, we followed the AT approach and compared hazard ratios (HRs) for GLP-1RA and DPP-4i initiators in terms of 3P-MACE, its components, and hospitalization for heart failure. We also performed an ITT sensitivity analysis within the same framework.

Additionally, we implemented the following supplementary and exploratory analyses: (1) comparison of HRs for all cardiovascular endpoints in subgroups stratified by pre-existing CVD; (2) comparison of HRs for the primary outcome (3P-MACE) in subjects who were female vs. male, aged 65 or older vs. 64 or younger, with claims-based diabetes duration above or below the median (91 months), treated vs. untreated with long-acting insulin, treated vs. untreated with sulfonylureas, treated vs. untreated with statins, treated vs. untreated with ACE inhibitors or sartans; (3) comparison of DPP-4i versus human-based (liraglutide, dulaglutide) or exendin-based (exenatide, lixisenatide) GLP-1RA.

For all analyses, we used Cox regression to estimate hazard ratios and tested statistical significance at the 0.05 level.

## Results

### Patient disposition and characteristics

**Fig. 1:**
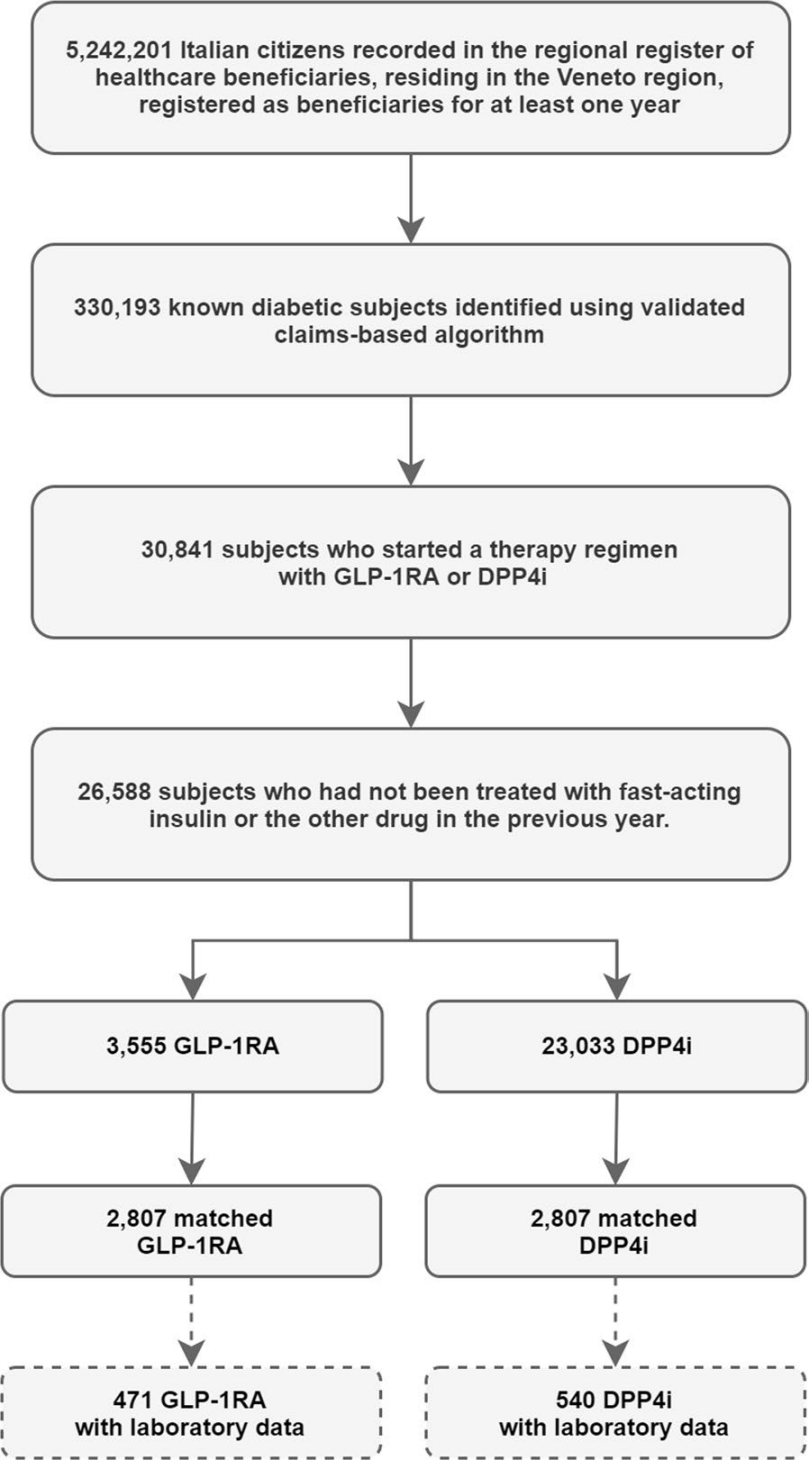
Study flowchart. Study framework with sample size

Starting from an initial pool of 5,242,201 Italian healthcare beneficiaries (Fig. 1), resident in Veneto, we found a diabetes diagnosis, via a validated claims-based algorithm [20], for 330,193 (6.3%) subjects. Of these, 30,841 were new GLP-1RA or DPP4i initiators. Exclusion of the patients who had been treated with fast-acting insulin yielded two groups of 3555 and 23,033 subjects, respectively, which were poorly balanced in terms of demographics, comorbidities, risk factors, and therapy (Table 1). We resolved this imbalance using PSM to obtain two cohorts of 2807 subjects each, who were matched for all variables (Additional file 1: Figure S1). Despite the pattern of utilization of GLP-1RA has changed over time [26], the balance was good also for what concerned index year distribution (Additional file 1: Table S2).

**Table 1:** Baseline clinical characteristics before and after matching

	Before matching				After matching			
	GLP-1RA (N = 3555)	DPP4i (N = 23,033)	SMD*	p value**	GLP-1RA (N = 2807)	DPP4i (N = 2807)	SMD*	p value**
Demographics								
Age at index date (years)	61.1 (9.7)	70.0 (10.6)	0.85	0	63.4 (8.6)	63.4 (10.2)	0.00	0.437
Female sex (%)	38.9	41.3	0.05	0.006	39.5	39.9	0.01	0.785
Claims-based history length^a^ (months)	50.3 (21.0)	44.0 (21.1)	0.30	0	47.8 (21.3)	48.3 (20.9)	0.02	0.242
Claims-based diabetes duration^b^ (months)	94.1 (61.5)	114.9 (67.3)	0.31	0.000	100.0 (62.8)	100.5 (61.8)	0.01	0.295
Risk factors								
Hypertension (%)	82.1	84.1	0.05	0.003	82.9	82.4	0.01	0.672
Dyslipidaemia (%)	67.9	71.7	0.08	0	68.8	69.0	0.00	0.908
Macrovascular complications								
Peripheral circulatory complications (%)	1.0	2.6	0.10	0	1.1	1.5	0.03	0.286
Infarction (%)	4.7	8.4	0.14	0	5.1	5.3	0.01	0.718
Ischemic heart disease (%)	9.1	14.6	0.16	0.000	9.7	10.2	0.02	0.532
Stroke or TIA (%)	3.1	5.6	0.11	0.000	3.2	3.9	0.04	0.169
Heart failure (%)	2.0	5.7	0.16	0	2.2	2.6	0.02	0.435
Cardiovascular disease (%)	13.1	22.2	0.22	0.000	13.7	15.1	0.04	0.149
Microvascular complications								
Neurological complications (%)	0.2	0.5	0.04	0.049	0.2	0.3	0.01	0.789
Ocular complications (%)	0.2	0.4	0.03	0.160	0.2	0.2	0.01	1.000
Renal complications (%)	0.2	0.8	0.07	0	0.2	0.3	0.01	0.789
Chronic kidney disease (%)	1.1	5.3	0.20	0.000	1.4	1.8	0.03	0.285
Severe hypoglycaemia (%)	0.5	1.2	0.07	0.000	0.4	0.6	0.02	0.570
Comorbidities								
Chronic pulmonary disease (%)	29.6	28.5	0.03	0.164	29.6	30.3	0.01	0.620
Systemic inflammatory disease (%)	2.2	1.9	0.02	0.414	2.2	2.3	0.00	1.000
Cancer (%)	8.9	14.0	0.15	0	10.3	9.8	0.02	0.563
Charlson comorbidity index	0.3 (0.9)	0.6 (1.3)	0.20	0	0.3 (0.9)	0.4 (1.0)	0.03	0.493
Glucose lowering medications								
No. of different A10B therapies^c^	1.8 (0.9)	1.8 (0.8)	0.03	0.201	1.8 (0.9)	1.8 (0.9)	0.02	0.306
Ever used insulin(%)	19.8	15.3	0.12	0	17.4	17.6	0.00	0.888
Long-acting insulin (%)	15.2	12.2	0.09	0	13.6	13.7	0.00	0.938
Metformin (%)	91.3	85.2	0.18	0	90.7	91.0	0.01	0.746
Sulfonylureas (%)	45.8	58.8	0.26	0	49.6	48.2	0.03	0.310
SGLT-2i (%)	6.2	0.8	0.44	0	2.2	1.3	0.07	0.015
Pioglitazone (%)	15.0	9.7	0.17	0	14.2	13.9	0.01	0.818
Other therapies								
ACE inhibitors (%)	71.8	71.6	0.00	0.838	72.0	72.2	0.00	0.905
Diuretics (%)	19.4	27.4	0.18	0.000	20.6	20.3	0.01	0.843
Beta blockers (%)	33.9	38.4	0.09	0.000	35.2	34.9	0.01	0.823
Other antihypertensives (%)	8.3	9.4	0.04	0.036	8.9	8.5	0.01	0.670
Statins (%)	59.1	64.0	0.10	0.000	60.5	61.0	0.01	0.702
Fibrates or omega-3 (%)	12.0	10.2	0.06	0.001	11.0	11.1	0.00	0.966
Ezetimibe (%)	2.6	2.0	0.04	0.017	2.4	2.6	0.02	0.606
Platelet aggregation inhibitors (%)	31.3	44.8	0.27	0.000	34.3	34.3	0.00	0.978
Clinical-laboratory data^d^								
Fasting glucose (mg/dL)					163.1 (44.8)	162.0 (50.4)	0.02	0.267
HbA1c (%)					7.8 (0.9)	7.7 (0.8)	0.11	0.095
Total cholesterol (mg/dL)					173.4 (36.6)	174.0 (36.4)	0.02	0.458
HDL cholesterol (mg/dL)					49.5 (13.4)	49.9 (13.1)	0.02	0.246
LDL cholesterol (mg/dL)					97.5 (31.7)	97.6 (32.5)	< 0.01	0.486
Triglycerides (mg/dL)					134.0 (53.6)	129.7 (56.7)	0.08	0.052
eGFR (mL/min/1.73 m^2^)					83.9 (16.6)	82.1 (18.5)	0.10	0.115
Systolic blood pressure (mm Hg)					142.2 (17.8)	141.8 (49.3)	0.01	0. 035
Diastolic blood pressure (mm Hg)					81.4 (10.0)	79.8 (10.4)	0.15	0.017

Therapy variables were calculated starting from 12 months before the index date, unless otherwise indicated. Pre-existing conditions were calculated with all available data up to the index date. Clinical-laboratory data refer to the visit closest to the index date. Absolute SMD values are shown

^a^ Time interval between the first available claim and the index date

^b^ Time interval between the first claim or exemption from co-payment indicating diabetes and the index date

^c^ Computed using all available data up to the index date

^d^ Data from 1011 available patients

* Standardized mean differences (positive if GLP-1RA greater).** Chi squared test for dichotomous variables (expressed as  %), Mann–Whitney’s U test otherwise

In the matched cohorts, GLP-1RA were distributed as follows: 43% liraglutide, 18% exenatide, 35% dulaglutide, 4% lixisenatide (semaglutide and albiglutide were unavailable in Italy); DPP-4i were distributed as follows: 45% sitagliptin, 23% vildagliptin, 14% alogliptin, 16% linagliptin, 2% saxagliptin. The average matched patient was 63 years old, had had diabetes for 8.3 years according to his or her claims, and had been treated with 1.8 classes of glucose-lowering drugs. More than half of the subjects were male (60%). Although most patients had hypertension (82%) and dyslipidemia (69%), only 15% had pre-existing CVD as of the index date. 91% were on metformin, 49% on sulfonylureas, and 14% were also on long-acting insulin. The use anti-hypertensive and lipid-lowering therapies was as high as in typical CVOTs.

Clinical-laboratory variables were available for 1011 subjects (18%), and were very well balanced within this subset, despite not having been used for PSM. Specifically, fasting glucose, HbA1c, total cholesterol, HDL cholesterol, LDL cholesterol, triglycerides, eGFR, and systolic blood pressure were adequately matched. Diastolic blood pressure exhibited a limited but statistically significant difference (Table 1).

### Cardiovascular outcomes

**Fig. 2:**
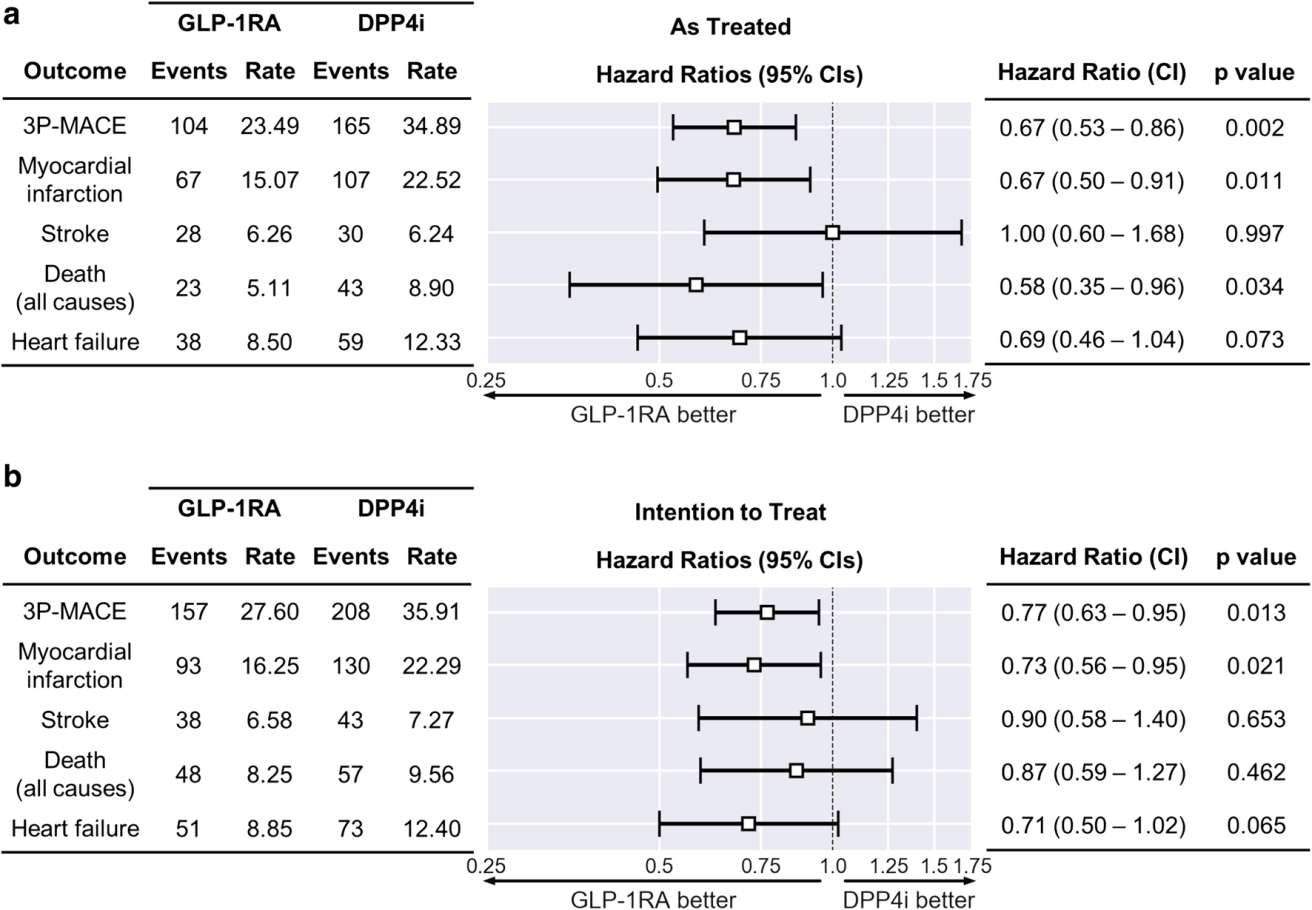
Outcome analysis. Results of Cox regression on primary and secondary outcomes in the AT (**a**) and ITT (**b**) dataset. Event rates are reported as number of events/1000 person-years

**Fig. 3:**
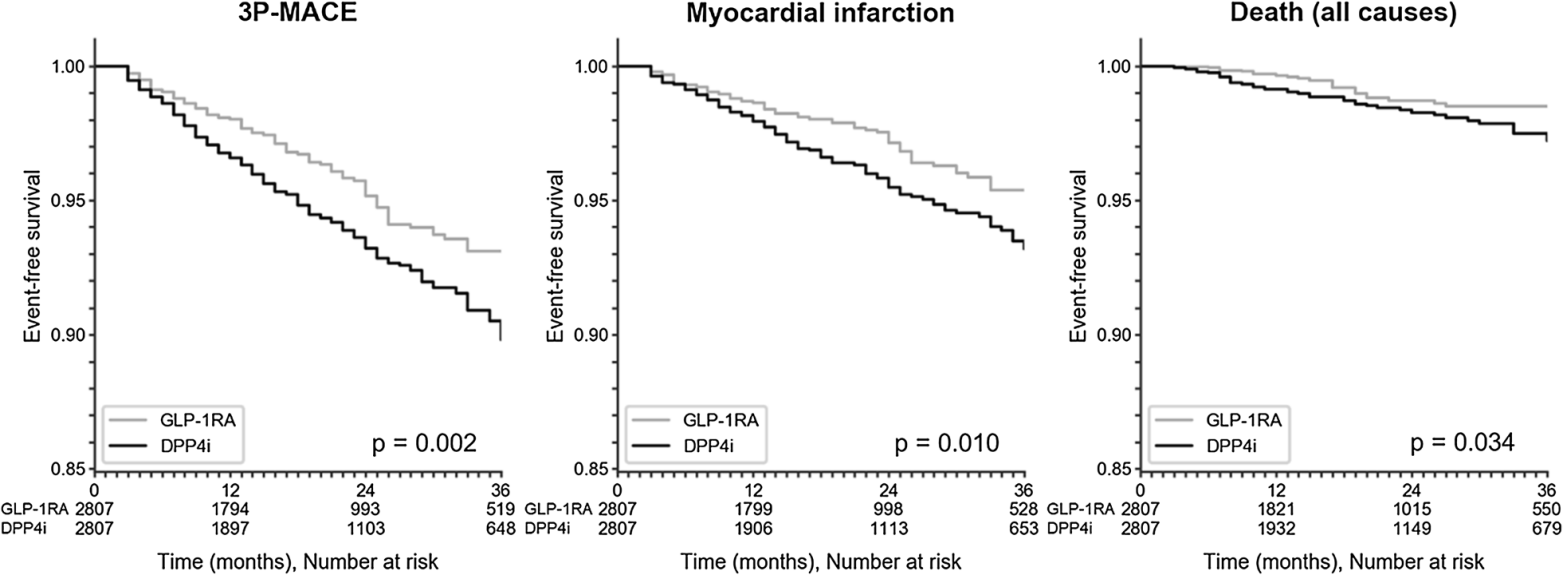
Kaplan–Meier curves. Event-free survival over time for statistically significant log-rank tests

The primary endpoint was the 3P-MACE, a composite outcome corresponding to the first occurrence of death, myocardial infarction, or stroke. The maximum follow-up length was 36 months: past that cutoff, fewer than 20% of the subjects remained in the study in the AT analysis. Median follow-up was 18 months (IQR 8–31) in the primary analysis, which increased to 28 months (IQR 14–36) in the ITT analysis. In the AT analysis, we observed 269 3P-MACE events with a rate of 29.4 events per 1000 person-years, 104 in the GLP-1RA cohort (23.5 events per 1000 person-years) and 165 in the DPP4i cohort (34.9 events per 1000 person-years). The HR associated with 3P-MACE was 0.67 (95% C.I. 0.53–0.86), significantly in favor of GLP-1RA initiators (p = 0.002). The rate of occurrence of all secondary endpoints was at least nominally lower in the GLP-1RA cohort, with the sole exception of stroke. The HRs associated with myocardial infarction (0.67; 95% C.I. 0.50–0.91; p = 0.011), and death (0.58; 95% C.I. 0.35–0.96; p = 0.034) were also significantly in favor of GLP-1RA (Fig. 2a). Kaplan–Meier curves are shown in Fig. 3.

The ITT sensitivity analysis yielded largely similar results for 3P-MACE (HR 0.77; 95% C.I. 0.63–0.95; p = 0.013) and myocardial infarction (HR 0.73, 95% C.I. 0.56 to 0.95, p = 0.021). Compared to the AT analysis, there was a loss of statistical significance for the death while the HR associated with stroke become nominally in favor of GLP-1RA initiators.

### Subgroup analyses

**Fig. 4:**
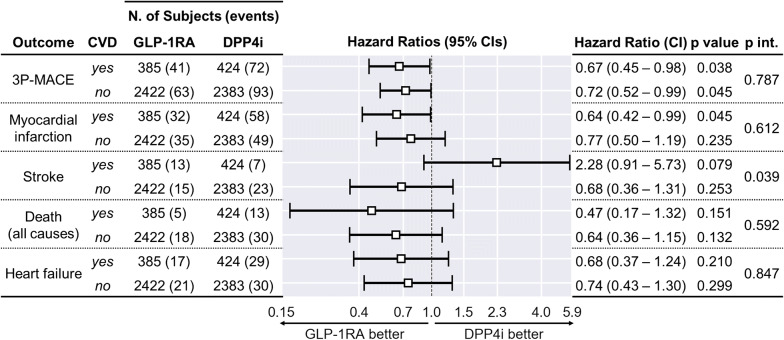
Outcome analysis by pre-existing CVD. Result of Cox regression on primary and secondary outcomes, after stratification according to pre-existing CVD. In the figure, “p int.” refers to the statistical significance testing on the interaction term (GLP-1RA or DPP4i × CVD yes or no) in the adjusted model

In the first subgroup analysis (Fig. 4), we compared HRs for all cardiovascular endpoints between the subgroups of patients with and without pre-existing CVD at the baseline. The majority of HRs were in favor of GLP-1RA vs. DPP-4i initiators regardless of pre-existing CVD. Stroke was a notable exception, favoring DPP-4i in the group with pre-existing CVD and GLP-1RA in the other, although not significantly in either case; the p-value for the interaction term in the adjusted model was 0.039. Statistically significant HRs favoring GLP-1RA initiators were associated with 3P-MACE in both subgroups (CVD HR: 0.67, 95% C.I. 0.45–0.98, p = 0.038; CVD-free HR: 0.72, 95% C.I. 0.52–0.99, p = 0.045), and myocardial infarction in the subgroup with pre-existing CVD (HR: 0.64, 95% C.I. 0.42–0.99, p = 0.038).

**Fig. 5:**
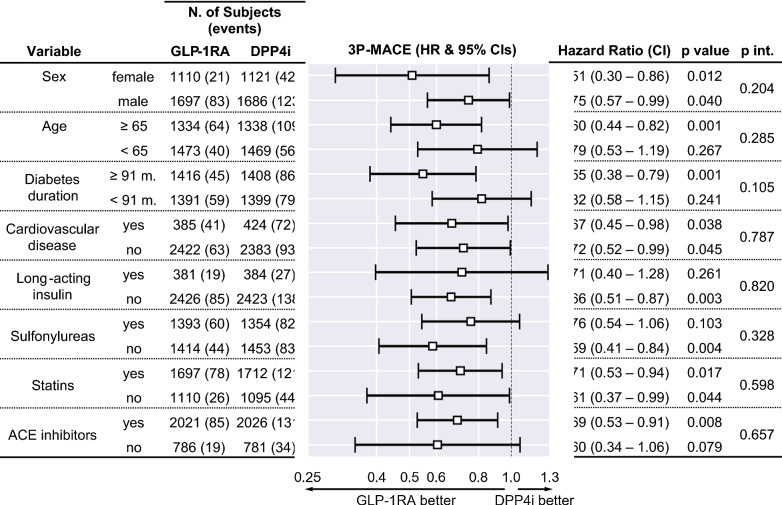
Sensitivity analysis for the primary outcome. Results of Cox regression on 3P-MACE, after stratification. In the figure, “p int.” refers to the statistical significance testing for the interaction term (GLP-1RA or DPP4i × stratification variable) in the adjusted model

In the second exploratory analysis (Fig. 5), we examined HRs for the primary outcome according to baseline characteristics. 3P-MACE HRs were significantly in favor of GLP-1RA irrespectively of sex, in those aged 65 years or older, with longer diabetes duration, not treated with long-acting insulin, not treated with sulfonylureas, treated with ACE inhibitors, but irrespectively of statin treatment. The p-values for the interaction terms were always > 0.05.

**Fig. 6:**
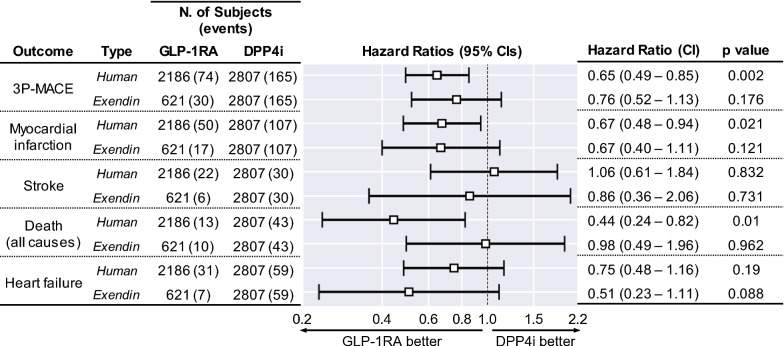
Sensitivity analysis based on GLP-1RA type. Results of Cox regression on primary and secondary outcomes, run separately for the comparison between human-based or exendin-based GLP-1RA versus DPP-4i

Finally, the outcome of DPP-4i initiators was compared to that of GLP-1RA initiators divided into human-based or exendin-based: although no substantial heterogeneity emerged in the comparison, initiators of human-based, but not exendin-based, GLP-1RA experienced lower rates of 3P-MACE, myocardial infarction, and all-cause death compared to DPP-4i initiators (Fig. 6).

## Discussion

### Summary of the findings

In this real-world retrospective study, we found that T2D patients who initiated a GLP-1RA exhibited a better cardiovascular outcome over a median of 18 months compared to similar patients who initiated a DPP-4i in the same period and healthcare setting. This effect was observed irrespective of sex and presence of established cardiovascular disease at baseline but was more pronounced in patients who were 65 years or older or had a longer disease duration.

### Study results in the context of available knowledge

Overall, this finding is in line with results of CVOTs on GLP-1RA and DPP-4i [27], which have been performed mostly in patients with established cardiovascular disease [28]. Remarkably, only 15% of patients in the matched cohorts of our study had established cardiovascular disease at baseline, reflecting a typical routine clinical care population. Therefore, our results indicate that the cardiovascular benefit of GLP-1RA over another commonly used glucose lowering strategy was observed in patients with a lower baseline cardiovascular risk compared to those enrolled in CVOTs.

Among components of the 3P-MACE, the lower rate of myocardial infarction was the main contributor to the better outcome observed in the GLP-1RA group. This finding is in agreement with a vast literature on the anti-atherosclerotic effects of GLP-1RA [29, 30]. In addition, the strong protection against myocardial infarction in this study population supports the hypothesis that the same anti-atherosclerotic mechanisms seen in so-called “secondary prevention” apply to patients who were mostly free from a history of cardiovascular events. However, we acknowledge that GLP-1RA could exert protective effects also by improving cardiac function either directly or by modulating epicardial fat [31, 32]. The difference in mortality rates between the two groups was significant only in the AT analysis, at least in part because of artificially inflated follow-up times arising from a lack of control with respect to drug discontinuation in the ITT analysis. As expected from epidemiological data [33], the incidence of stroke was much lower than that of myocardial infarction and no difference in stroke rates was observed between patients who received GLP-1RA and those who received DPP-4i. This finding is partially unexpected because GLP-1RA have shown a prominent capacity to reduce stroke compared to placebo [34], whereas no effect was reported for DPP-4i versus placebo [35]. Future real-world analyses on this topic will be of interest.

No difference was observed in the rates of heart failure between groups. This was expected because trial data indicate that GLP-1RA exert minor protective effects against heart failure [36], while DPP-4i are mostly neutral [37], with the sole possible exception of saxagliptin, which was under-represented in pur study.

### Human versus exendin-based GLP-1RA

It should be noted that not all GLP-1RA have demonstrated cardiovascular protective effects in dedicated CVOTs. In patients with T2D and a recent acute coronary event, no difference was observed in the rates of MACE between lixisenatide and placebo [38] and the trial testing once weekly exenatide showed non-significant reductions in 3P-MACE versus placebo [39]. With regards to the latter, it is of interest that the combination with SGLT-2 inhibitors may potentiate the cardiovascular protection exerted by once weekly exenatide [40]. Yet, it has been speculated that cardiovascular protection might be conveyed selectively by human-based GLP-1RA (liraglutide, semaglutide, dulaglutide, albiglutide) and not by exendin-based GLP-1RA (exenatide and lixisenatide). A meta-analysis reported a nearly significant (p = 0.06) interaction between structural origin of GLP-1RA and the HR for 3P-MACE [36]. In our study, 77.8% of patients in the GLP-1RA group was receiving a human-based molecule. The rates of 3P-MACE were significantly lower versus DPP-4i only for human-based GLP-1RA and not for exendin-based GLP-1RA, with such difference being attributable entirely to all-cause death. It should be noted that the number of patients on exendin-based GLP-1RA was small and the between-group balance was no longer guaranteed in this subgroup analysis. Specifically, as shown in Table S3, while balance was retained in the human-based GLP-1RA vs. DPP4i comparison, some variables were unbalanced in the exendin-based GLP-1RA vs. DPP4i comparison (including age, claims-based diabetes duration, Charlson comorbidity index, usage of long-acting insulin). Therefore, further studies on the comparison of cardiovascular effectiveness between human- and exendin-based GLP-1RA are needed to confirm or discard our finding.

In a previous observational study using 2006–2013 data from a large US commercial database, no difference was observed in the rates of cardiovascular events in patients initiating a GLP-1RA or a DPP-4i during 1 year follow-up [41]. However, in such study, > 70% of patients were on exenatide [41]. Therefore, our new results are not in contrast with prior real-world evidence and indirectly support the hypothesis that human-based GLP-1RA may be endowed with more pronounced cardiovascular protective effects than exendin-based GLP-1RA.

### Study limitations and strength

This study has limitations related to its retrospective non-randomized design. Patients who initiate GLP-1RA or DPP-4i typically differ in many clinical characteristics and the resulting confounding by indication would hamper comparative assessment. We thus used PSM to obtain a pseudo-randomised condition, characterised by equal a posteriori probabilities of matched subjects being assigned to either treatment given baseline covariates. Although matching variables were constructed from administrative claims, we checked matching quality using clinical-laboratory data available for a subset of patients in the database. In addition to providing a snapshot of glucose, pressure, and lipid control in the population, this strategy verified that matching on claims-based variables forced balance into other clinical variables that could affect the outcome. Other relevant variables were not available, including compliance, lifestyle, and socio-economic status, but the balance achieved in laboratory data, even if they were not used for PSM, is reassuring of the successful matching procedure.

The study has notable strengths. During the study period, the two treatment strategies were equally positioned in the treatment algorithm, could only be prescribed by diabetes clinics, and we included only patients who initiated either class of drugs in the same period and in the same geographic area, without being previously treated with the other class. Together with matching on diabetes duration and history of GLM, this helped minimizing immortal time bias and time lag bias [42]. Finally, follow-up duration was longer than in other studies of the same type [41], thereby providing medium-term comparative information.

## Conclusion

In the absence of direct comparative trials, our real-world data confirm findings of network meta-analysis [27] that GLP-1RA may be more effective than DPP-4i in protecting patients with T2D against cardiovascular events. Remarkably, a better cardiovascular outcome was observed after initiation of GLP-1RA versus DPP-4i in patients with a baseline cardiovascular risk that was much lower than in CVOTs. These data call for a more widespread use of GLP-1RA in routine clinical practice.

## Supplementary information

**Additional file 1.** Supplementa data.

## Data Availability

The datasets used and analyzed during the current study are available from the corresponding author on reasonable request.

## Abbreviations

AT: As treated
CVD: Cardiovascular disease
CVOT: Cardiovascular outcome trial
DPP-4i: Dipeptidyl peptidase 4 inhibitors
eGFR: Estimated glomerular filtration rate
GLM: Glucose lowering medications
GLP-1RA: Glucagon like peptide-1 receptor agonist
HR: Hazard ratio
ITT: Intention to treat
MACE: Major adverse cardiovascular events
PS: Propensity score
PSM: Propensity score marching
rHIE: Regional Health Information Exchange
SGLT2i: Sodium glucose cotransporter-2 inhibitors
T2D: Type 2 diabetes
TIA: Transient ischemic attack
