# Supplementary material for: Better cardiovascular outcomes of type 2 diabetic patients treated with GLP-1 receptor agonists versus DPP-4 inhibitors in clinical practice
Source: Cardiovasc Diabetol. 2020 Jun 10;19:74. doi: 10.1186/s12933-020-01049-w (PMC7288543; doi:10.1186/s12933-020-01049-w)
Supplement: Supplementary file 1 — Additional file 1. Supplementa data. [file 12933_2020_1049_MOESM1_ESM.docx]

**Additional file**

**Figure S1. Balance before and after matching.** Absolute standardized mean differences before and after PSM. The 0.10 threshold is indicated by a dashed line.


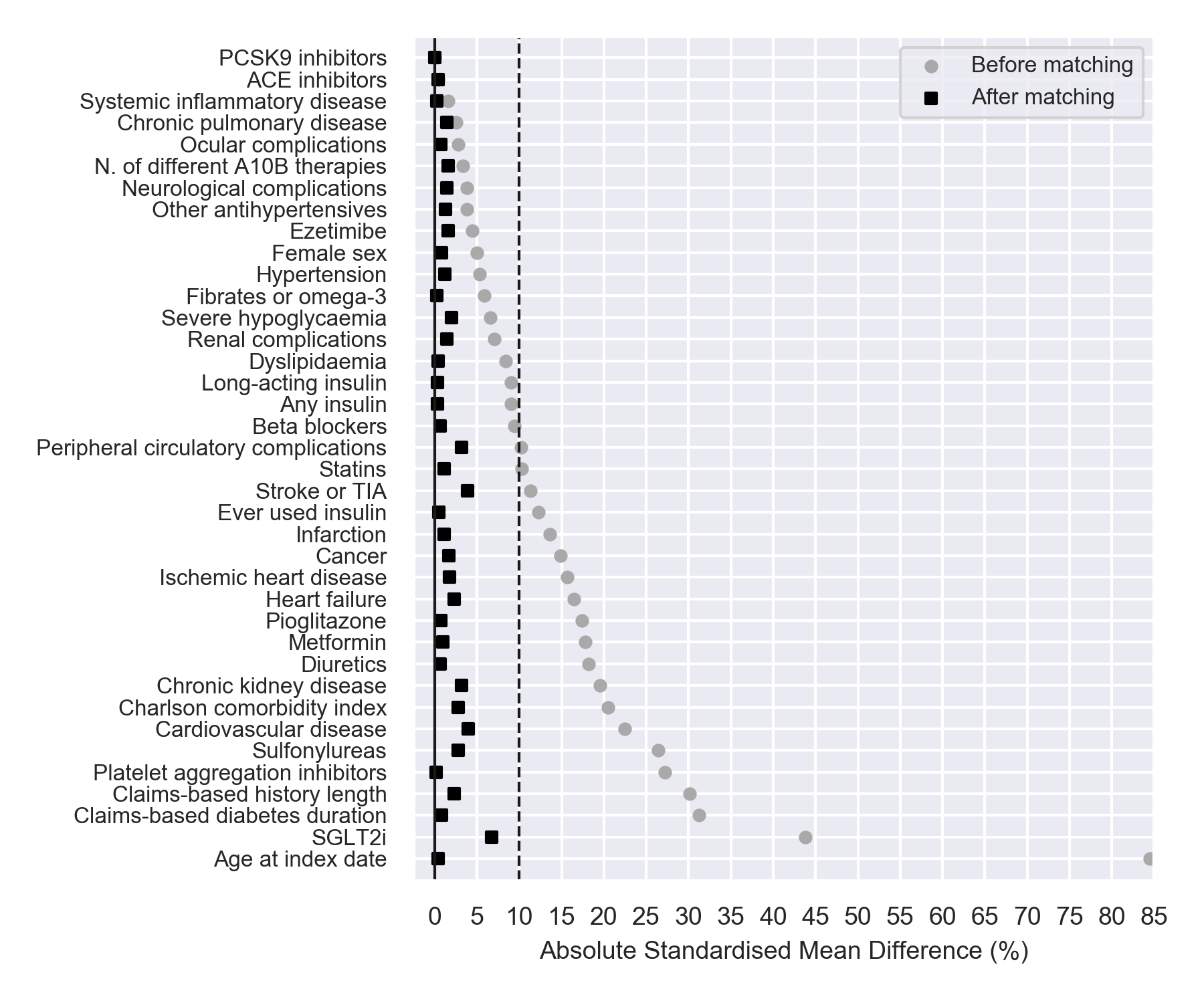


**Table S1**. **Claims-based definition of study variables**. Each variable was defined as the presence of at least one of the claims-based indicators in the corresponding row. Medication names have been internally mapped 1:1 to ATC codes, exemptions from copayment to regional exemption codes.

| **Variable** | **ICD-9-CM Diagnosis Codes** | **ICD-9-CM Procedure Codes** | **Medications** | **Exemptions from Copayment** |
| --- | --- | --- | --- | --- |
| **Hypertension** | 401-405 |  | ACE inhibitors, diuretics, beta blockers, other antihypertensives | Hypertension |
| **Dyslipidaemia** |  |  | Statins, fibrates, omega 3, PCSK9 inhibitors, ezetimibe | Dyslipidaemia |
| **Peripheral circulatory complications** | 440, 250.7 | 00.55, 38.48, 39.50, 39.52, 39.71, 39.90 |  |  |
| **Myocardial Infarction** | 410-414 |  |  |  |
| **Ischemic heart disease** | 410-414 | 00.66, 36.03, 36.06, 36.07, 36.10, 36.19 |  | Ischemic heart disease |
| **Stroke or TIA** | 431-436 | 00.61-00.65 |  | Stroke or TIA |
| **Heart failure** | 428 |  |  | Heart failure |
| **Cardiovascular disease** | 410-414, 428, 431-436, 440 | 00.55, 00.61-00.66, 36.03, 36.06, 36.07, 36.10, 36.19, 38.48, 39.50, 39.52, 39.71, 39.90 |  | Ischemic heart disease, stroke or TIA, heart failure |
| **Neurological complications** | 250.6 |  |  |  |
| **Ocular complications** | 250.5 |  |  |  |
| **Renal complications** | 250.4 |  |  |  |
| **Chronic kidney disease** | 585-587 |  |  | Chronic kidney disease |
| **Severe hypoglycaemia** | 250.3, 250.8, 250.10, 250.12 |  |  |  |
| **Chronic pulmonary disease** | 491-496, 518.83, 518.84 |  | Medications for obstructive airway diseases | Chronic pulmonary disease, asthma |
| **Systemic inflammatory disease** |  |  |  | Rheumatoid arthritis, systemic lupus erythematosus, connective tissue disease, Sjogren's syndrome, psoriasis, ankylosing spondylitis |
| **Cancer** |  |  |  | Cancer |

**Table S2**. **Index year distribution in the matched cohort**. Columns 2 and 3 report the number of matched GLP-1RA or DPP4i initiators (respectively) whose index date was in the year indicated by column 1.

| **Index year** | **GLP-1RA** | **DPP4i** |
| --- | --- | --- |
| **2011** | 1 | 0 |
| **2012** | 200 | 136 |
| **2013** | 377 | 433 |
| **2014** | 329 | 292 |
| **2015** | 374 | 517 |
| **2016** | 525 | 495 |
| **2017** | 614 | 586 |
| **2018** | 387 | 348 |

**Table S3. Baseline clinical characteristics of human-based GLP-1RA and exendin-based GLP-1RA initiators vs. DPP4i initiators.** Therapy variables were calculated starting from 12 months before the index date, unless otherwise indicated. Pre-existing conditions were calculated with all available data up to the index date. Clinical-laboratory data refer to the visit closest to the index date. Absolute SMD values are shown.

|  | **Human-based GLP-1RA** | | | | **Exendin-based GLP-1RA** | | | |
| --- | --- | --- | --- | --- | --- | --- | --- | --- |
|  | **GLP-1RA (N=2186)** | **DPP4i (N=2807)** | **SMD^*^** | **p value^**^** | **GLP-1RA (N=621)** | **DPP4i (N=2807)** | **SMD^*^** | **p value^**^** |
| **Demographics** |  |  |  |  |  |  |  |  |
| **Age at index date (years)** | 63.8 (8.6) | 63.4 (10.2) | 0.03 | 0.063 | 62.1 (8.4) | 63.4 (10.2) | -0.14 | 0.001 |
| **Female sex (%)** | 38.8 | 39.9 | -0.02 | 0.448 | 42 | 39.9 | 0.04 | 0.359 |
| **Claims-based history length^a^ (months)** | 49.3 (22.1) | 48.3 (20.9) | 0.05 | 0.01 | 42.4 (17.0) | 48.3 (20.9) | -0.29 | 0 |
| **Claims-based diabetes duration^b^ (months)** | 102.0 (63.4) | 100.5 (61.8) | 0.02 | 0.267 | 93.0 (60.1) | 100.5 (61.8) | -0.12 | 0.002 |
| **Risk factors** |  |  |  |  |  |  |  |  |
| **Hypertension (%)** | 82.8 | 82.4 | 0.01 | 0.773 | 83.3 | 82.4 | 0.02 | 0.654 |
| **Dyslipidaemia (%)** | 69.3 | 69 | 0.01 | 0.845 | 67.1 | 69 | -0.04 | 0.393 |
| **Macrovascular complications** |  |  |  |  |  |  |  |  |
| **Peripheral circulatory complications (%)** | 1.2 | 1.5 | -0.02 | 0.482 | 0.8 | 1.5 | -0.06 | 0.275 |
| **Infarction (%)** | 5.5 | 5.3 | 0.01 | 0.827 | 3.5 | 5.3 | -0.08 | 0.084 |
| **Ischemic heart disease (%)** | 9.9 | 10.2 | -0.01 | 0.756 | 8.9 | 10.2 | -0.04 | 0.353 |
| **Stroke or TIA (%)** | 3.4 | 3.9 | -0.02 | 0.444 | 2.3 | 3.9 | -0.09 | 0.064 |
| **Heart failure (%)** | 2.3 | 2.6 | -0.02 | 0.537 | 2.1 | 2.6 | -0.03 | 0.555 |
| **Cardiovascular disease (%)** | 14 | 15.1 | -0.03 | 0.312 | 12.6 | 15.1 | -0.07 | 0.119 |
| **Microvascular complications** |  |  |  |  |  |  |  |  |
| **Neurological complications (%)** | 0.2 | 0.3 | -0.01 | 0.915 | 0.2 | 0.3 | -0.02 | 0.91 |
| **Ocular complications (%)** | 0.2 | 0.2 | 0 | 0.886 | 0.2 | 0.2 | -0.02 | 0.963 |
| **Renal complications (%)** | 0.3 | 0.3 | 0 | 0.842 | 0 | 0.3 | -0.06 | 0.383 |
| **Chronic kidney disease (%)** | 1.5 | 1.8 | -0.02 | 0.527 | 1 | 1.8 | -0.06 | 0.202 |
| **Severe hypoglycaemia (%)** | 0.5 | 0.6 | -0.02 | 0.726 | 0.3 | 0.6 | -0.03 | 0.641 |
| **Comorbidities** |  |  |  |  |  |  |  |  |
| **Chronic pulmonary disease (%)** | 29.9 | 30.3 | -0.01 | 0.805 | 28.7 | 30.3 | -0.04 | 0.455 |
| **Systemic inflammatory disease (%)** | 2.3 | 2.3 | 0 | 0.937 | 2.1 | 2.3 | -0.01 | 0.893 |
| **Cancer (%)** | 10.3 | 9.8 | 0.02 | 0.531 | 10 | 9.8 | 0.01 | 0.925 |
| **Charlson comorbidity index** | 0.4 (0.9) | 0.4 (1.0) | 0 | 0.203 | 0.2 (0.8) | 0.4 (1.0) | -0.11 | 0.03 |
| **Glucose lowering medications** |  |  |  |  |  |  |  |  |
| **No. of different A10B therapies^c^** | 1.8 (0.9) | 1.8 (0.9) | 0.03 | 0.225 | 1.7 (0.8) | 1.8 (0.9) | -0.04 | 0.37 |
| **Ever used insulin (%)** | 18.3 | 17.6 | 0.02 | 0.553 | 14.3 | 17.6 | -0.09 | 0.06 |
| **Long-acting insulin (%)** | 14.9 | 13.7 | 0.04 | 0.231 | 8.9 | 13.7 | -0.14 | 0.001 |
| **Metformin (%)** | 90.3 | 91 | -0.03 | 0.381 | 92.4 | 91 | 0.05 | 0.295 |
| **Sulfonylureas (%)** | 50.2 | 48.2 | 0.04 | 0.181 | 47.7 | 48.2 | -0.01 | 0.831 |
| **SGLT-2i (%)** | 2.7 | 1.3 | 0.1 | 0.001 | 0.5 | 1.3 | -0.08 | 0.122 |
| **Pioglitazone (%)** | 13.5 | 13.9 | -0.01 | 0.723 | 16.4 | 13.9 | 0.07 | 0.123 |
| **Other therapies** |  |  |  |  |  |  |  |  |
| **ACE inhibitors (%)** | 71.9 | 72.2 | -0.01 | 0.861 | 72.3 | 72.2 | 0 | 0.989 |
| **Diuretics (%)** | 20.1 | 20.3 | 0 | 0.904 | 22.1 | 20.3 | 0.04 | 0.356 |
| **Beta blockers (%)** | 35.3 | 34.9 | 0.01 | 0.79 | 34.9 | 34.9 | 0 | 0.975 |
| **Other antihypertensives (%)** | 8.6 | 8.5 | 0 | 0.955 | 9.8 | 8.5 | 0.05 | 0.334 |
| **Statins (%)** | 61.3 | 61 | 0.01 | 0.821 | 57.3 | 61 | -0.07 | 0.1 |
| **Fibrates or omega-3 (%)** | 11.2 | 11.1 | 0 | 0.995 | 10.6 | 11.1 | -0.02 | 0.78 |
| **Ezetimibe (%)** | 2.4 | 2.6 | -0.01 | 0.684 | 2.3 | 2.6 | -0.02 | 0.722 |
| **Platelet aggregation inhibitors (%)** | 36.2 | 34.3 | 0.04 | 0.169 | 27.9 | 34.3 | -0.14 | 0.002 |

^*^ Standardized mean differences (positive if GLP-1RA greater).^**^ Chi-squared test for dichotomous variables (expressed as %), Mann-Whitney’s U test otherwise. ^a^ time interval between the first available claim and the index date. ^b^ time interval between the first claim or exemption from co-payment indicating diabetes and the index date. ^c^ computed using all available data up to the index date.
